# Stem cell treatments and Parkinson's disease: Science and misconceptions

**DOI:** 10.1177/1877718X261434672

**Published:** 2026-04-17

**Authors:** Athena Stamper, Harry Bulstrode, Roger A Barker

**Affiliations:** 1 Cambridge Stem Cell Institute, University of Cambridge, Cambridge, UK; 2 Department of Clinical Neurosciences, John van Geest Centre for Brain Repair, University of Cambridge, Cambridge, UK

**Keywords:** cell therapy, stem cell biology, neurosurgery, clinical trials, preclinical studies, misconceptions, Parkinson's disease

## Abstract

Parkinson's disease (PD) is a common neurodegenerative disorder associated with the accumulation of alpha-synuclein in Lewy bodies and Lewy neurites, as well as cell loss, including ventral midbrain dopaminergic (vmDA) neurons in the substantia nigra. This degeneration is responsible for some of the characteristic motor features of PD, which, in the early stages of the disease, can be successfully treated pharmacologically. However, in later stages of the disease, the efficacy of these drugs declines, and they can cause side effects. Dopamine cell therapy, which involves replacing lost vmDA neurons by implanting new exogenous ones, is a promising alternative. While research on dopamine cell therapies has made substantial progress over the last few decades, from early foetal transplants to stem cell-derived transplants currently in clinical trials, it is also susceptible to potential misconceptions. This review summarises the past, present, and future of this therapeutic strategy whilst also discussing these potential misconceptions. In doing so, the status of dopamine cell therapy for PD will be critically summarised.

## Plain language summary

Stem Cell Treatments and Parkinson's disease: Science and Misconceptions

Parkinson's disease involves the loss of dopamine-producing cells in the brain that are important for controlling movement. As a result patients develop movement problems such as shaking, stiffness, and slowness. In the early stages of the disease these symptoms can often be managed well with medication. Over time, however, medication tends to become less effective and may cause unwanted side effects. One potential alternative approach is to replace the lost dopamine-producing cells by transplanting new ones into the brain. Research in this area has been ongoing for several decades, starting with transplants using dopamine cells from foetal tissue and more recently with transplants using dopamine cells made from stem cells, which are now being tested in clinical trials. This review looks at how this approach has developed in the past, where it currently stands, and what it may offer in the future. Whilst doing so it also discusses potential misconceptions associated with this approach to set realistic expectations and highlight the true potential of this approach fostering confidence and optimism for its future.

## Introduction

Parkinson's disease (PD), named after the English physician James Parkinson, who first described the condition in 1817 in his Essay on the Shaking Palsy,^1^ is a common neurodegenerative disease. PD involves, in part, the progressive loss of ventral midbrain A9 dopaminergic (vmDA) neurons projecting primarily from the substantia nigra to the striatum, resulting in a marked reduction in striatal dopamine levels. This loss is thought to be associated with the misfolding and aggregation of the alpha-synuclein protein, resulting in the formation of cytoplasmic inclusions: Lewy bodies within cell bodies, and Lewy neurites within neuronal processes.^2^ Since the nigrostriatal pathway is important for controlling aspects of movement, this degeneration is associated with some of the characteristic motor features of PD, which include bradykinesia (slowness of movement), rigidity and resting tremor.^3^ However, the pathology of PD is not confined to vmDA neurons but extends to various neuronal populations within the central, autonomic, and enteric nervous systems, giving rise to a broad range of non-motor symptoms including cognitive impairment leading to dementia, sleep disturbances, constipation, autonomic dysfunction, psychiatric issues and loss of smell (anosmia).^3^ Nevertheless, the loss of vmDA neurons and the resulting motor abnormalities are the primary targets of most current PD treatments.

Current pharmacological treatments focus on restoring dopamine levels in the striatum. Administration of the dopamine precursor L-DOPA has remained the mainstay of this treatment for over 50 years, with dopamine agonists and inhibitors of dopamine metabolism also being important agents. However, intermittent oral administration of L-DOPA leads to fluctuating levels in the plasma and brain. In the early stages of PD, surviving vmDA neurons buffer these fluctuations by regulating dopamine storage, release, and reuptake. However, as these neurons are progressively lost, these fluctuations cannot be buffered as effectively, leading to motor complications in later stages of PD. These include wearing off, unpredictable on-off fluctuations, and excessive movements such as L-DOPA-induced dyskinesias (LIDs). All of these motor complications can initially be managed by manipulating the dose and timings of L-DOPA administration as well as introducing agents that target the LIDs themselves, such as amantadine. Nevertheless, more invasive treatments are eventually needed, such as jejunal or subcutaneous infusions of L-DOPA or apomorphine (a dopamine agonist), or neurosurgical treatments, including MRI-guided focused ultrasound lesioning or deep-brain stimulation (DBS). However, each has its own benefits and risks.

Dopamine cell therapy, involving the transplantation of exogenous vmDA neurons, typically into the striatum itself, has long offered the promise of restoring striatal dopamine levels in a targeted, physiological manner, minimising the off-target effects associated with pharmacological treatment. While research on dopamine cell therapy has made substantial progress over the last several decades, it has also faced many challenges, which have led to a number of potential misconceptions about the approach, including its safety, scalability, mechanism of action, efficacy, and its ability to offer a significant therapeutic advantage in this condition. This review aims to summarise this therapeutic strategy-past, present, and future-whilst also discussing some of the misconceptions above.

## Past – human foetal transplants

One of the early promising dopamine cell therapies for PD involved the transplantation of developing vmDA neurons obtained from the ventral mesencephalon of human foetuses (hfVM neurons) collected from terminated pregnancies. This approach was based on the pioneering preclinical work demonstrating that fVM neurons from rat foetuses (rfVM neurons), collected at the final stages of their normal development (embryonic day 13–14), could survive, engraft and release dopamine when transplanted into the striatum of rats with unilateral nigrostriatal dopaminergic lesions induced by the injection of the neurotoxin 6-hydroxydopamine (6-OHDA).^4–7^ Such transplantation was also associated with improvements in motor deficits characteristic of these lesioned animals, such as drug-induced rotation. These findings were subsequently replicated when 6-OHDA rats were transplanted with hfVM neurons^8^ (of the same developmental stage as the rfVM neurons), allowing the commencement of open-label clinical trials in the late 1980s.^9–19^ In these allogeneic hfVM transplant trials, patients were placed on immunosuppressive regimens similar to those used in solid organ transplantation, consisting of several immunosuppressive agents initiated at the time of transplantation and maintained for a limited period afterwards to prevent rejection. These early trials yielded variable results, but in the best cases, patients experienced significant, prolonged improvements in their clinical motor scores, which were associated with an increase and, in some cases, normalised uptake of the radiolabelled ligand ^18^F-DOPA in the striatum on positron emission tomography (PET) imaging. These improvements even allowed some patients to discontinue their dopaminergic medications. These particularly successful cases may contribute to a potential misconception that “**dopamine cell therapy for PD can be a cure**”. However, this therapy will never be curative as it does not target the underlying neuropathology. Instead, it aims only to repair and restore this pathologically affected system to normal, and thus is only a symptomatic treatment.

Nonetheless, these encouraging findings, in concert with the end of a ban in the USA preventing federal funding for research using human foetal tissue, allowed for the commencement of two National Institutes of Health (NIH)-funded double-blind placebo-controlled trials in the 1990s.^20,21^ Unfortunately, improvements in clinical motor scores were variable, and a notable number of patients (15% and 56%, respectively) developed graft-induced dyskinesias (GID) (involuntary movements driven by the graft in the absence of medication), which in some cases necessitated further neurosurgical intervention with DBS. The origin of these GIDs is still unclear, but the most likely explanation is that they are due to the presence of unwanted serotonergic neurons that were co-grafted^22,23^ - cells that develop close to the hfVM in the brainstem. Post-mortem analyses additionally revealed the presence of α-synuclein containing Lewy body and Lewy neurite–like structures within neurons of the graft, suggesting that they may acquire PD pathology.^24,25^ Whilst this was only seen after 10 years, and then in the minority of neurons^26^ it highlighted another potential misconception that “**a dopamine cell therapy for PD may succumb to the disease process and as such is not a useful treatment**”. However, it takes many years for such pathology to emerge, and even in the longest surviving transplant that has come to post mortem 24 years after the graft surgery, the majority of vmDA neurons in the graft did not have any such alpha synuclein pathology.^27^

Inevitably, these disappointing clinical trial outcomes led to waning interest in dopamine cell therapy, especially as DBS was being shown to be successful in clinical trials.^28^ Subsequent re-evaluation of these early trials, however, identified several important limitations in their design, including patient selection.^29^ The importance of patient selection was largely overlooked at the time of the early trials, when PD was predominantly viewed as a more homogenous disease, leading to the potential misconception that “**all patients with PD could be suitable for dopamine cell therapy**”. Since then, it has become clear that PD is a heterogeneous disease, and differences in both disease stage and clinical presentation affect patients’ suitability for cell therapy. Indeed, younger patients in the early stages of PD with less advanced disease and a clear response to L-DOPA treatment were seen to achieve better outcomes than those in later stages who had developed more significant LIDs. This may be because degeneration of vmDA neurons is less extensive, helping preserve downstream circuitry into which transplanted neurons can integrate.

In light of this re-evaluation, a new multicentre (Lund and Cambridge) study, TRANSEURO, commenced in 2010 with two aims. The first was to conduct an observational study examining the natural history of younger-onset, early-stage PD patients - those viewed as ideal candidates for dopamine cell therapy.^30^ The second was to conduct an open-label trial of hfVM transplantation.^31^ In the observational study, patients demonstrated slow disease progression without the development of significant LIDs or cognitive impairment, confirming their suitability for dopamine cell therapy. On this basis, patients were then randomly selected from this observational study for inclusion in the transplantation study, with the primary end point being the change in UPDRS part III defined off motor scores 3 years post-transplantation. Again, the improvements following hfVM transplantation were variable. This variability was thought to be in part linked to the centre where the transplant surgery was performed, with those transplanted in Lund showing greater improvements in clinical motor scores than those transplanted in Cambridge (although sample sizes were too small to allow statistical confirmation). One potential reason for this centre-dependent variability is the fact that different neurosurgical devices were used at each centre. Indeed, the Rehncrona–Legradi (R–L) device was used in Lund; however, as it was not Conformité Européenne (CE) marked, it could not be used in Cambridge. Consequently, a modified version of the device was used instead. Another potential reason for this variability was that patients in Lund were, on average, younger and had less advanced disease than those in Cambridge.

Although these factors could, in principle, be addressed in future trials, TRANSEURO ultimately demonstrated that hfVM transplants are not logistically feasible due to persistent difficulties in obtaining sufficient tissue. To obtain adequate material for transplantation, the tissue used in this trial spanned a range of developmental stages, and the time it spent in hibernation media prior to processing and transplantation varied and the final tissue implant could not be standardised either between or within patients, as each individual received tissue from three separate foetuses per hemisphere. In some cases, tissue availability remained insufficient, leading to the cancellation of 87 planned surgeries during the trial. This shortage of tissue also prevented the use of higher doses (tissue from four or more separate foetuses in each hemisphere) of hfVM, which may have produced greater improvements in clinical motor scores. An alternative to hfVM transplantation that avoids its associated logistical issues is the use of stem cell–derived transplants, which, owing to recent advances in vmDA neuron derivation, have become a highly promising option.

## Present – pluripotent stem cell derived transplants

Pluripotent stem cells (PSCs) possess two defining characteristics: the ability to self-renew indefinitely and to differentiate into any cell type of the body, including vmDA neurons. PSCs include embryonic stem cells (ESCs) and induced PSCs (iPSCs). ESCs are derived from the inner cell mass of blastocyst-stage embryos, raising ethical issues in some jurisdictions. In contrast, iPSCs are generated by reprogramming somatic cells through the forced expression of pluripotency-associated transcription factors (typically Oct3/4, Sox2, Klf4, and c-Myc), thereby avoiding these ethical issues. Additionally, iPSCs can be autologous and, thus, cells derived from them avoid immune rejection issues, unlike those derived from allogeneic iPSCs or ESCs. However, compared with ESCs, iPSC reprogramming potentially increases the risk of genetic or epigenetic abnormalities. In the absence of consensus on whether to use ESCs or iPSCs, both are being used to derive vmDA neurons in ongoing clinical trials.

Early efforts to differentiate PSCs into vmDA neurons typically involved forming neural intermediates that expressed the PAX6 marker. This was typically achieved through the spontaneous aggregation of PSCs into embryoid bodies^32^ or through the co-culture of PSCs with stromal feeder cells^33–39^ or astrocytes.^40^ Whilst these protocols produced DA neurons, as evidenced by TH expression, they were not specifically vmDA neurons. Therefore, it is not surprising that they showed poor survival when transplanted into 6-OHDA-lesioned rodents, resulting in little to no motor improvement. A significant breakthrough came following the observation that vmDA neurons instead arise from neural intermediates expressing the markers FOXA2 and LMX1, prompting the development of new differentiation protocols.^41,42^ Early protocols relied on the dual inhibition of SMAD signalling pathways, namely TGF-β and BMP, combined with activation of SHH and canonical WNT signalling pathways, with the addition of appropriate growth factors.^43^ These neurons showed superior survival and function when transplanted into 6-OHDA-lesioned mice compared to neurons differentiated using earlier protocols. Since then, several additional protocols have been developed, optimising the timing and concentration of growth factors^44,45^ and incorporating flow-sorting techniques.^46–48^

These protocols have now been adapted to comply with good manufacturing practice (GMP) standards,^49–52^ allowing PSC-derived vmDA neurons to transition to early-stage clinical trials to investigate their safety and efficacy when transplanted as progenitors. However, many remain hesitant about this transition. This hesitancy may be due to a potential anxiety that “**PSC-based cell therapy may be unsafe in the long term and, thus, not suitable for clinical use at this time**”. This is largely because the transplantation of even a small proportion of undifferentiated PSCs can lead to teratoma formation. Indeed, it has been shown that transplantation of hESC-derived vmDA progenitors containing as little as 1% of undifferentiated hESCs results in teratoma formation in a subset of immunodeficient mice.^53^ Therefore, safety concerns surrounding the use of PSCs are not unfounded. Nevertheless, it is important to recognise that, with rigorous preclinical testing and strict quality control, PSC-based cell therapies can be developed to meet the safety standards needed for clinical use. This is highlighted by the fact that at least 1200 patients have been transplanted with PSC-based cell therapies for various indications worldwide, without tumour formation to date.^54^ Such testing typically involves ensuring that the proportion of undifferentiated PSCs, assessed by the expression of pluripotency markers such as OCT3/4 and NANOG, is less than 0.1% - a threshold at which teratoma formation is not observed. Indeed, it has been shown that transplantation of hESC-derived vmDA progenitors containing 0.1% undifferentiated hESCs does not result in teratoma formation in immunodeficient mice, at least within the short-term follow-up of study.^53^ Such testing has been performed for all progenitors used in ongoing clinical trials, all of which have not been shown to contain detectable numbers of hPSCs.^49,52,53,55^

In 2020, the first patient transplantation of PSC-derived vmDA progenitors was published.^56^ This transplantation was carried out in a single male patient using autologous iPSC-derived vmDA progenitors. While there were some improvements in ^18^F-DOPA uptake and clinical motor scores, the most dramatic response was in quality of life, with the patient reporting a significant improvement. However, as only one patient was treated, these results could not be generalised, highlighting the need for early-stage clinical trials involving multiple patients.

Since then, several such trials have been initiated across the USA, Europe, and Asia (see Table 1), including our own STEM-PD trial, a joint effort between Cambridge (UK) and Lund (Sweden). At the time of writing, results have been published from three of these trials in peer-reviewed journals. One such trial was a phase I/II, open-label trial in Japan where patients received either a low or a high dose of allogeneic iPSC-derived vmDA progenitors.^57^ No evidence of abnormal growth or tumour formation was observed in any patient and there were no reports of GIDs, consistent with the hypothesis that contaminating 5-HT neurons caused these side effects in earlier hfVM trials. However, given the relatively short follow-up periods of these trials, long-term safety concerns cannot yet be fully excluded at this stage. PET imaging revealed a modest increase in ^18^F-DOPA uptake in all patients, with greater increases in the high-dose group. However, absolute ^18^F-DOPA uptake values in the high-dose group were lower than those in the low-dose group, and values in both groups remained well below those in healthy individuals. This suggests that the number of transplanted progenitors that survive and mature into functional dopaminergic neurons remains well below the number needed to fully repair this network.^58^ Interestingly, this parallels what was seen in the TRANSEURO trial, where the results were again variable, but the patient who showed the greatest improvement in clinical motor scores also demonstrated restoration of ^18^F-DOPA uptake to levels comparable to those of healthy controls.^31^

**Table 1.** Ongoing and planned clinical trials investigating stem cell-derived dopamine cell therapy for Parkinson's disease.

Trial	Country	Patient cohort	Cell source/dose	Immuno-suppression	Status of trial
**Academic Led Trials**					
CiRA (Phase I/II open label trial) **jRCT2090220384**	Japan	N = 7 (3 females and 4 males). Age range = 50–69.	Fresh allogenic iPSC-derived vmDA progenitors (homozygous for the most prevalent HLA haplotype in the Japanese population). Low dose = 2.1–2.6 million cells per hemisphere. High dose = 5.3–5.5 million cells per hemisphere.	Tacrolimus	All patients transplanted. Preliminary data published.
STEM-PD (Phase I open label trial) **NCT05635409**	UK/Sweden	N = 8. Age range = 50–75.	Cryopreserved ESC-derived vmDA progenitors. Low dose = 3.54 million cells per hemisphere. High dose = 7.08 million cells per hemisphere	Basiliximab, tacrolimus (or cyclosporine if tacrolimus is not tolerated), azathioprine, and steroids.	All patients transplanted.
Massachusetts General Hospital (Phase I open label trial) **NCT06687837**	USA	N = 8 (Estimated). Age range = 45–80.	Autologous iPSC-derived vmDA progenitors. Low dose = 4 million cells per hemisphere. High dose = 8 million cells per hemisphere.	Undisclosed.	Ongoing.
McLean Hospital (Phase I open label trial) **NCT06422208**	USA	N = 6 (Estimated). Age range = 55–80.	Cryopreserved autologous iPSC-derived vmDA progenitors.	Undisclosed.	Ongoing.
University of California, San Diego **NCT06482268**	USA	N = 7 (Estimated). Age range = 40–75.	Allogenic iPSC-derived vmDA progenitors (under the name (CT1-DAP001). Dose = 4.2–5.4 million cells per hemisphere.	Undisclosed.	Ongoing.
**Company Led Trials**					
S. Biomedics (Phase I/IIa open label trial) **NCT05887466**	South Korea	N = 12 (3 females and 9 males). Average age = 60.3.	Fresh ESC-derived vmDA progenitors. Low dose = 3.15 million cells. High-dose = 6.30 million cells.	Basiliximab, methyl-prednisolone/ prednisolone, and tacrolimus.	All patients transplanted. Preliminary data published.
iRegene Therapeutics (Phase I open label trial) **NCT06608355**	China	N = 6 (Estimated). Age range = 18–70.	Allogenic iPSC-derived vmDA progenitors (under the name NouvNeu001).	Undisclosed.	Ongoing.
iRegene Therapeutics (Phase I/II open label trial) **NCT06167681**	China	N = 40 (Estimated). Age range = 50–75.	Allogenic iPSC-derived vmDA progenitors (under the name NouvNeu001).	Undisclosed.	Ongoing.
Nuwacell Biotechnologies (Phase I open label trial) **NCT06978920**	China	N = 48 (Estimated). Age range = 40–75.	Allogenic iPSC-derived vmDA progenitors (under the name NCR201).	Undisclosed.	Ongoing.
XellSmart Biomedical (Open label trial) **NCT06145711**	China	N = 3 (Estimated). Age range = 30–70.	Autologous iPSC-derived vmDA progenitors.	Undisclosed.	Ongoing.
iCamuno Biotherapeutics (Phase I open label trial) **NCT06821529**	China	N = 12 (Estimated). Age range = 39–75.	Autologous iPSC-derived vmDA progenitors. Dose = 4 million cells per hemisphere.	Undisclosed.	Ongoing.
Shanghai UniXell Biotechnology (Phase I open label trial) **NCT06778265**	China	N = 12 (Estimated). Age range = 50–75.	Autologous iPSC-derived vmDA progenitors (under the name UX-DA001).	Undisclosed.	Ongoing.
ASPIRO - Aspen Neuroscience (Phase I/IIa open label trial) **NCT06344026**	USA	N = 9 (Estimated). Age range = 45–80.	Autologous iPSC-derived vmDA progenitors (under the name ANPD001).	Undisclosed.	Ongoing.
Bluerock Therapeutics/Bayer (Phase I open label trial) **NCT04802733**	USA	N = 12 (3 females and 9 males). Median age = 67.	Cryopreserved ESC-derived vmDA progenitors (under the name Bemdaneprocel). Low dose = 0.9 million cells per hemisphere. High dose = 2.7 million cells per hemisphere.	Basiliximab, methyl-prednisolone, and tacrolimus.	All patients transplanted. Preliminary data published.
exPDite-2 - Bluerock Therapeutics/Bayer (Phase III double blind randomised trial) **NCT06944522**	USA	N = 102 (Estimated). Age range = 45–75.	Cryopreserved ESC-derived vmDA progenitors (under the name Bemdaneprocel).	Undisclosed.	Ongoing.
Sumitomo Pharma (PhaseI/II double blind randomised trial) **NCT06753331**	USA	N = 23 (Estimated). Age range = 40–70.	Allogenic iPSC-derived vmDA progenitors (under the name DSP-1083). Dose = 2.7 million cells per hemisphere.	Undisclosed.	Ongoing.
Oryon (Phase I open label trial) **NCT06422208**	USA	N = 6 (Estimated). Age range = 55–80.	Cryopreserved autologous iPSC-derived vmDA progenitors.	Undisclosed.	Ongoing.
Kenai Therapeutics (Phase Ib/IIa open label trial) **NCT07106021**	USA	N = 12 (Estimated). Age range = 45–75.	Allogenic iPSC-derived vmDA progenitors (under the name RNDP-001).	Undisclosed.	Ongoing.

Complementing this trial, two further Phase I/IIa open-label trials were conducted in the USA (Bluerock Therapeutics/Bayer)^59^ and South Korea^60^ and have been published in which patients were transplanted with either a low or a high dose of hESC-derived vmDA progenitors. Similar to the Japanese trial, the absence of abnormal growth, tumour development, or GIDs suggested favourable short-term safety, while modest improvements in clinical motor scores pointed toward potential efficacy, but again without normalisation of ^18^F-DOPA uptake on PET.

It must be noted that all three trials had several limitations, including a small sample size, the lack of a control group, and an open-label design in which both patients and investigators were aware of the treatment they were receiving. Therefore, efficacy data from these trials must be interpreted with caution as it could be subject to a placebo effect and/or observer bias. Nevertheless, the short-term safety data from these studies have given regulatory authorities confidence to allow larger clinical trials. Indeed, Bluerock Therapeutics/Bayer has now launched a phase III, randomised, sham-surgery-controlled, double-blind trial that commenced in September 2025. This trial will be significantly larger than any previous phase I/II trials and is designed to rigorously assess the efficacy of hESC-derived vmDA progenitor transplantation under more controlled and unbiased conditions, with the hope that it will lead to licensing if results are positive. Similarly, a larger follow-up trial to the Japanese trial is under consideration.

## Future

**Figure 1.**
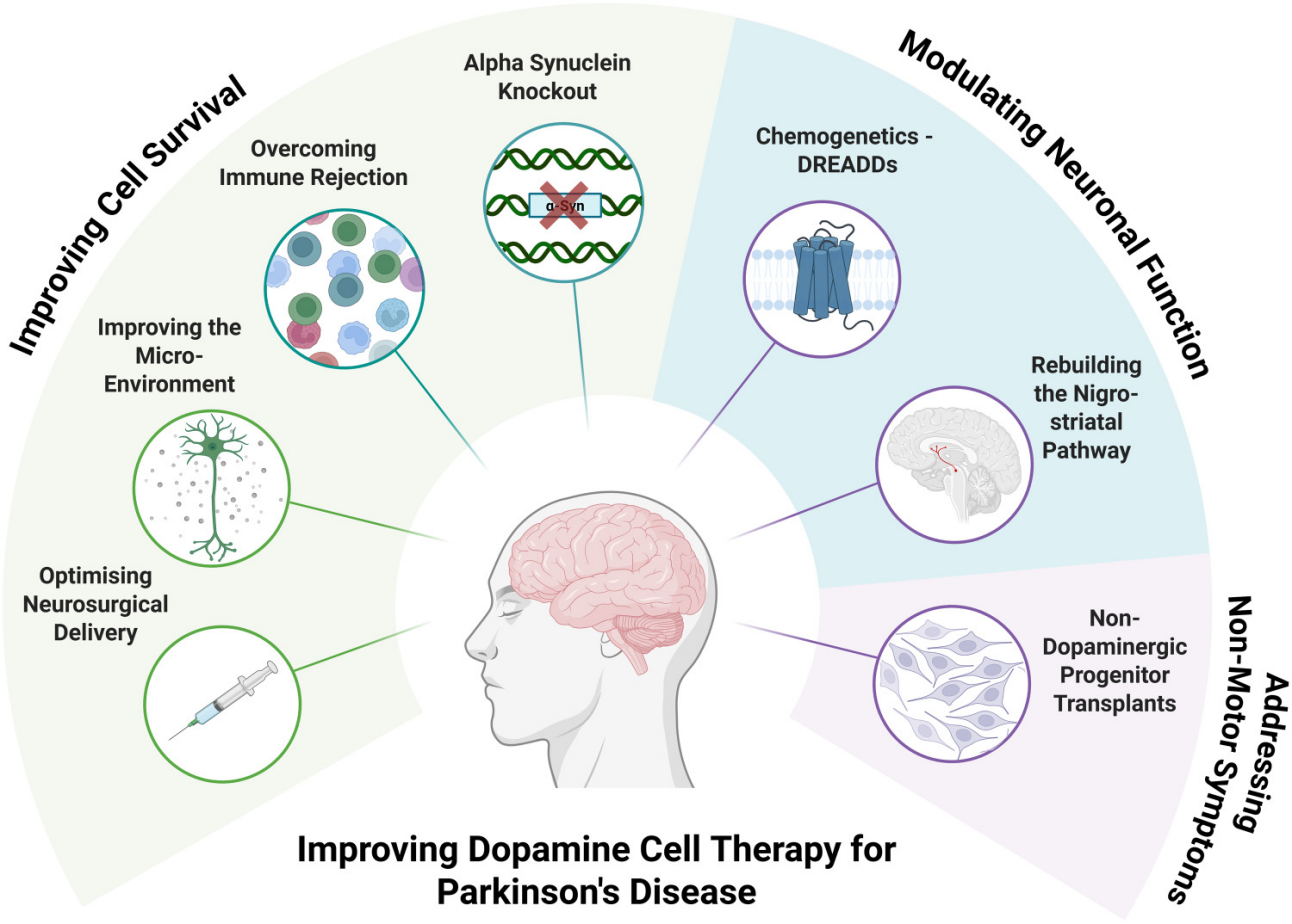
Improving stem cell-derived dopamine cell therapy for Parkinson's disease.

Overall, preliminary data from early-stage clinical trials of PSC-derived vmDA progenitor transplants indicate that, similar to outcomes observed with hfVM transplants, clinical benefit remains small and variable.^57,59,60^ Although larger phase III trials are now being considered or indeed started (see above), some view this as being premature, as no patients in the previous trials have demonstrated normalisation of F-DOPA uptake on PET, reductions in PD medication or significant improvements in clinical motor scores.^61^ Thus, there is a need to optimise cell dose, delivery, and the immunosuppression regimens used before larger, more definitive trials can be conducted. Without undertaking smaller studies in this iterative fashion, there is a risk of perpetuating a potential misconception that “**dopaminergic cell therapies are incapable of competitively delivering on their initial therapeutic promise**”. Therefore, it is more important than ever to consider how shortcomings evident in the current approaches could be surmounted to prevent abandoning this therapeutic approach (Figure 1).

### Improving progenitor survival

One of the biggest shortcomings of current approaches is poor progenitor survival, as illustrated by the US trial, which based its dosing on the assumption that only about 5% of the transplanted progenitors would survive.^59^ A seemingly straightforward strategy to address this issue is to increase the initial dose of transplanted progenitors. However, the delivery of these progenitors to the human brain currently relies on straight-needle neurosurgical devices, which often require multiple passes to distribute them across the required brain volumes.^62^ Since these progenitors are delivered to subcortical regions, each pass must first traverse several centimetres of overlying brain. With each additional pass increasing the risk of vascular injury by an estimated 1%, it is unsurprising that, for example, two patients in the TRANSEURO trial^31^ and one patient in the South Korean trial^60^ experienced intracerebral haemorrhages. Therefore, increasing progenitor doses is difficult to justify if it requires more passes. Beyond these safety concerns, current neurosurgical devices are also subject to reflux up the needle, severely restricting the speed and volume of delivery and compromising the viability of the delivered progenitors. Taken together, these limitations underscore the pressing need for neurosurgical devices that provide better target coverage through a single pass while preventing reflux. Such advances would not only support the delivery of higher progenitor numbers but also increase the proportion that survive.

Unlike the developing brain, which is rich in neurotrophic factors that support progenitor survival and maturation, the adult brain into which these progenitors are transplanted is a much less permissive environment, especially in the context of PD and neurosurgical trauma. Therefore, even if surgical delivery is optimised, this environment remains a fundamental barrier to progenitor survival. To address this limitation, neurotrophic factors could be delivered following transplantation. Glial cell line-derived neurotrophic factor (GDNF) is the most promising candidate, as extensive preclinical research demonstrates its ability to support the survival of endogenous vmDA neurons.^63^ Therefore, preclinical research has since explored whether it can similarly improve the survival of transplanted vmDA progenitors, with promising findings. For example, delivery of GDNF prior to, but not after, transplantation of hESC-derived vmDA progenitors has been shown to increase survival,^64^ although this was also associated with decreased maturation. Therefore, the timing of GDNF delivery needs to be refined to both optimise survival whilst mitigating any negative effects. In addition to the lack of neurotrophic support, there is evidence that the graft itself can further worsen the local microenvironment by releasing the pro-inflammatory cytokine tumour necrosis factor-α (TNF-α), which contributes to cell death.^65^ Suppressing TNF-α signalling with adalimumab, a clinically approved inhibitor used to treat various autoimmune conditions, has also been shown to increase the survival of hESC-derived vmDA progenitors after transplantation.

Even with optimised surgical delivery and strategies to improve the microenvironment, the survival of transplanted progenitors ultimately depends on their ability to evade immune rejection. Because the brain is relatively immune-privileged (at least after restoration of blood-brain barrier integrity post-transplantation), these progenitors may be less prone to rejection than those of solid organs. Furthermore, these progenitors are known to have relatively low levels of Human Leukocyte Antigen (HLA) expression.^66^ However, current in vivo and in vitro models for assessing immunogenicity are limited, making it difficult to accurately predict the immune response. Therefore, immunosuppressive regimes comparable to those used for solid organ transplants are typically used, which are associated with significant risks, including increased susceptibility to infection. Therefore, methods to reduce or even overcome the need for immunosuppression are highly desirable.

Administration of autologous progenitors would be one such way to obviate the need for immunosuppression.^56^ However, the time, cost, and labour required to generate patient-specific iPSCs for the derivation of these progenitors are not insubstantial.^67^ The resulting progenitors may also be at increased risk of acquiring alpha-synuclein pathology, given that the donor somatic cells would likely contain genes associated with PD. In monogenic cases, correction of the underlying PD-causing mutation is, in theory, straightforward. However, the vast majority of PD cases are sporadic and linked to numerous genetic variants that cannot be corrected before the generation of autologous progenitors. An alternative to autologous progenitors is HLA-matched progenitors. In the Japanese trial, the iPSC line used to derive progenitors was homozygous for the most prevalent HLA haplotype in the Japanese population; thus, some patients were, by chance, HLA-matched to the graft. This allowed a reduced-intensity immunosuppressive regimen to be used - a decision backed by preclinical findings.^68^ Extending this approach to enable all patients to be HLA-matched would instead involve establishing a PSC bank containing multiple allogeneic lines that collectively represent the HLA diversity of the host population. It has been estimated that around 150 hESC lines would be required to serve the UK population.^69^ The number needed for other countries varies depending on the degree of genetic variability within their populations. Generating banks with this number of lines would require screening a significant number of potential donors, making it both costly and time-consuming. Furthermore, line-to-line variability in vmDA neuronal differentiation potential would necessitate protocol optimisation for each individual line.

Another alternative focuses on generating hypoimmunogenic PSC lines. This has been accomplished by knocking out key HLA genes and overexpressing the transmembrane protein CD47, which plays a central role in reducing immunogenicity.^70^ These modified cells retain the properties of normal PSCs and can successfully differentiate into endothelial cells, smooth muscle cells, and cardiomyocytes. Notably, when transplanted into fully HLA-mismatched mice, they evade immune rejection, providing proof of concept that hypoimmunogenic PSCs can be used to generate vmDA neurons for transplantation without immunosuppression. Such cells have been used to derive hypoimmunogenic islet cells,^71^ which are currently being investigated in humans as a potential cell therapy for type 1 diabetes.^72^ However, because CD47 is also frequently overexpressed in many cancer types,^73^ enabling them to evade immune detection, there remains a risk that if transplanted progenitors undergo malignant transformation, CD47 expression could similarly shield them from immune detection. Such risks can be mitigated by introducing a “safety switch” into these modified PSCs, allowing selective ablation of any rapidly proliferating cells.^74^

### Modulating neuronal activity

A further shortcoming of current cell therapy approaches is that progenitors are transplanted directly into the striatum, thereby preventing appropriate reconstruction of the upstream circuitry vital for the normal physiological control of neuronal activity. Transplanting these progenitors into the substantia nigra may instead allow such reconstruction. A key challenge, however, lies in promoting the long-distance axonal growth required to re-establish downstream circuitry of the nigrostriatal pathway. Emerging evidence suggests that targeted direct current electrical stimulation may support this process; for example, human fetal midbrain dopaminergic explants from the ventral tegmental area have been shown to orient and grow toward the cathode during such stimulation.^75^

Nevertheless, even with appropriate circuit reconstruction, there may still be circumstances, such as suboptimal clinical benefit or ongoing disease progression, in which a means of externally modulating neuronal activity would be valuable. Chemogenetics, which involves genetically introducing receptors engineered to respond exclusively to an inert ligand into a cell population of interest, offers a potential means for this type of adjustment. Whilst several different engineered receptors exist, the so-called designer receptors exclusively activated by designer drugs (DREADDs), which were initially developed by modifying human muscarinic receptors to respond to clozapine-N-oxide (CNO), remain the most commonly used.^76^ A subset of these DREADDs are coupled to the Gq signalling pathway, which in neurons typically results in depolarisation and increased excitability, as originally demonstrated in hippocampal cells in mice.^77^ Such depolarisation and increased excitability have also been demonstrated in vmDA neurons and are associated with increased dopamine release and motor improvements following transplantation into 6-OHDA-lesioned rodents.^78–81^ An additional advantage of chemogenetics is that DREADD-expressing neurons can be selectively visualised using radiolabelled ligands for PET imaging, such as [^11^C]CNO^82^ - an important corollary to the current ^18^F-DOPA or PE2i PET imaging. However, the genetic introduction of DREADDs raises concerns about the risk of introducing deleterious mutations. Introducing a “safety switch” into these cells may again be a way to mitigate these risks.^74^

### Addressing non-motor symptoms

One final shortcoming is that current cell therapy approaches address only some motor features of PD, while doing little to address the non-motor symptoms that have an equally profound impact on a patient's quality of life. Therefore, it is important to consider how cell therapies could also be used to address non-motor symptoms resulting from the degeneration of neuronal populations beyond vmDA neurons. Addressing these non-motor symptoms may instead require transplanting complementary cell types alongside vmDA progenitors. In particular, degeneration of the basal forebrain cholinergic neurons has been shown to correlate with some of the cognitive deficits and dementia of PD, and thus represents an intriguing target for future cell replacement.^83^

## Conclusion

Dopamine cell therapy research has progressed considerably, evolving from early hfVM transplants to the current clinical trials of PSC-derived vmDA progenitors. However, this research may also be shaped by several misconceptions. By addressing these potential misconceptions, this review aims to set realistic expectations and highlight the true potential of dopamine cell therapy, fostering confidence and optimism for its future.
